# Metabolism along the life journey of T cells

**DOI:** 10.1093/lifemeta/load002

**Published:** 2023-01-19

**Authors:** Min Peng, Ming O Li

**Affiliations:** Department of Basic Medical Sciences, School of Medicine, Tsinghua University, Beijing 100084, China; Institute for Immunology, Tsinghua University, Beijing 100084, China; Tsinghua-Peking Center for Life Sciences, Beijing 100084, China; Beijing Key Laboratory for Immunological Research on Chronic Diseases, Tsinghua University, Beijing 100084, China; Immunology Program, Sloan Kettering Institute, Memorial Sloan Kettering Cancer Center, New York, NY 10065, USA; Immunology and Microbial Pathogenesis Program, Weill Cornell Graduate School of Medical Sciences, Cornell University, New York, NY 10065, USA

**Keywords:** T cells, immunometabolism, glycolysis, OXPHOS, FAO, acetyl-CoA

## Abstract

T cells are one of few cell types in adult mammals that can proliferate extensively and differentiate diversely upon stimulation, which serves as an excellent example to dissect the metabolic basis of cell fate decisions. During the last decade, there has been an explosion of research into the metabolic control of T-cell responses. The roles of common metabolic pathways, including glycolysis, lipid metabolism, and mitochondrial oxidative phosphorylation, in T-cell responses have been well characterized, and their mechanisms of action are starting to emerge. In this review, we present several considerations for T-cell metabolism-focused research, while providing an overview of the metabolic control of T-cell fate decisions during their life journey. We try to synthesize principles that explain the causal relationship between cellular metabolism and T-cell fate decision. We also discuss key unresolved questions and challenges in targeting T-cell metabolism to treat disease.

## Introduction

**Figure 1:**
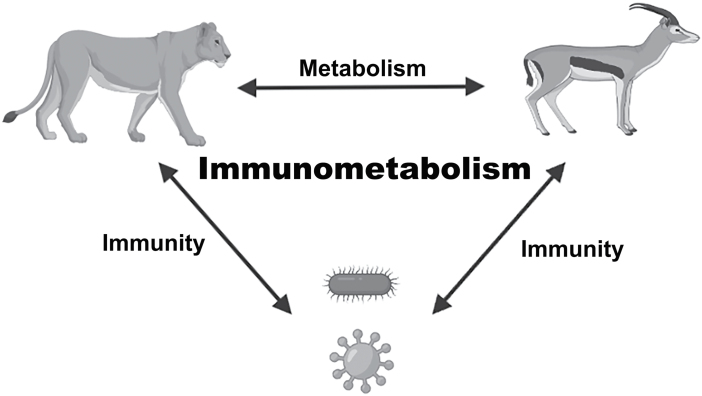
An evolutionary perspective of immunometabolism. Metabolism and immunity are two strong selective forces during natural selection. Inter- and intra-species competition for limited food resource drives optimization of the metabolic network to the highest efficiency of energy usage. Both predator and prey cannot compete with infectious pathogens by conventional flight or fight responses, and thus a robust immune system is the key to survival from infections. The extensive cross-regulation between metabolism and immunity ensures that both systems work efficiently to confer survival advantage during evolution.

Cellular metabolism is a defining feature of all living organisms with the regulation of the anabolism of biomolecules supporting organismal growth and the catabolism of such molecules to generate cellular energy antagonizing the principle of increased entropy, which otherwise causes the demise of all life. During evolution, the predator-prey relationship among different species drives continuous optimization of the metabolic network in the direction of highest efficiency of resource usage. However, both predator and prey cannot compete with infectious pathogens by fighting or fleeing, and cells of the immune system have evolved to deal with the challenge. Thus, metabolism and immunity are two prominent forces that drive natural selection, which lays the foundation for a cross-regulation between them (Fig. 1). Indeed, the past three decades have witnessed the development of the immunometabolism field that has revealed the reciprocal regulation of organismal metabolism and the immune system. Although immune cells play important roles in metabolic organs and regulate metabolic diseases [1, 2], we herein focus on how metabolic processes regulate the behavior of T cells, a pivotal component of the adaptive immune system.

The core of cell metabolism are the extraction of bioenergy (i.e. catabolism), assembly of biomolecules as cellular and molecular building blocks (i.e. anabolism), and the maintenance of a proper redox balance. Catabolic metabolism, such as glycolysis and fatty acid oxidation, produces energy, while anabolic metabolism generates building blocks required for cell growth and proliferation. Proper redox balance is of paramount importance in metabolism because many biochemical reactions involve the transfer of electrons that if not properly regulated can lead to generation of reactive species that can cause pathological cellular and tissue damage. Of note, the basic metabolic pathways were elucidated between the 1930s and 1960s, the golden era of classical biochemistry studies, during which post-mitotic cells (such as liver extracts) and tumor cell lines were used to define enzyme activities and metabolic flows. One of the few studies using primary immune cells to investigate metabolism was Otto Warburg’s demonstration that activated leukocytes engage in aerobic glycolysis (i.e. fermentation in the presence of oxygen, also called the Warburg effect) instead of mitochondrial oxidative phosphorylation (OXPHOS) [3], a metabolic feature akin to his earlier observations in tumor cells [4]. Although biochemical reactions are identical among different cell types, the activities of these biochemical reactions can be highly heterogeneous in different cell types. Indeed, the immunometabolism field has not identified novel biochemical reactions that only happen in immune cells, and all metabolic pathways studied in immune cells also exist in other cell types. It is the adaptation of certain metabolic pathways during immune responses that makes immunometabolism a new and important sub-field.

## Points of consideration for T-cell metabolism-focused research

T-cell responses play central roles in the host defense against infections and cancer, and they trigger pathology autoimmune in nature. Activated T cells divide every 4–8 h [5, 6], which makes them one of the fastest proliferating cell types in mammals. The dramatic clonal expansion of activated T cells must be supported by nutrient uptake and their metabolism in cells, which leads to the question of what role does cellular metabolism play in T-cell responses [7]. With extensive studies in the past decades, metabolic pathways involved in T-cell responses have been broadly characterized. Excellent reviews about immunometabolism (including T-cell immunometabolism) have been published recently [1, 2, 8–11]. Thus, we direct the reader to these reviews to explore the basic knowledge and concepts in immunometabolism. Before diving into the detailed metabolic regulation of T-cell responses, we need to consider several key questions about immunometabolism in general.

### Metabolic regulator vs. metabolic enzyme

Cellular metabolism is the collection of catabolic and anabolic biochemical reactions that take place in cells, most of which are catalyzed by enzymes. In lower organisms, such as bacteria, metabolism is largely cell-autonomous and depends on nutrient availability. Mammalian cells have evolved complex signaling pathways to sense and respond to the fluctuation of nutrients in the environments, such as mammalian target of rapamycin (mTOR) and AMP-activated protein kinase (AMPK) pathways [12]. These interconnected signaling pathways form a network to adjust cellular metabolism according to cellular needs and nutrient availability. All these signaling pathways play important roles in regulating T-cell metabolism and function, which have been elegantly reviewed [13, 14]. However, the targets of these kinases are not limited to cell metabolism, and they control a broad spectrum of cellular responses independent of metabolism, including transcriptional, translational, and post-translational events that are essential for most (if not all) cell types, including T cells. To what extent these pleiotropic signaling pathways regulate T-cell responses through modulating metabolism remains incompletely understood.

Enzymatic reactions directly affect the concentration of specific metabolites in cells. T-cell phenotypic changes upon inhibition or activation of a specific enzyme can be explained by the reduction of substrate and/or the increase of product of a specific biochemical reaction. Although certain metabolic enzymes possess non-metabolic (i.e. ‘moonlighting’) functions that have been proposed to play important roles in T cells [15–17], the authentic and usually core function of metabolic enzymes is to change concentrations of metabolites in cells, which will be our focus of discussion in this review.

### Driver vs. passenger

T-cell responses are accompanied by metabolic changes, like all other cellular processes. A key unsettled question in the field is whether the observed metabolic alteration is the cause or consequence of a T-cell phenotypic change. For example, naïve T cells engage OXPHOS for energy production [18], but it is unknown whether engaging OXPHOS is the cause or consequence of naïve T-cell quiescence. In many cases, loss of naïve T-cell quiescence is coupled with metabolic activation. For example, depletion of tuberous sclerosis 1 (TSC1), a negative regulator of mTOR complex 1 (mTORC1) signaling, causes loss of naïve T-cell quiescence in association with metabolic activation [19]. It remains unknown whether metabolic activation of TSC1-deficient T cells is due to T-cell activation or whether it causes T-cell activation, since hyperactivation of mTORC1 in TSC1-deficient T cells may induce both responses. On the other hand, mice with T-cell-specific transgenic overexpression of the glucose transporter GLUT1 show spontaneous T-cell activation and signs of autoimmunity at late stages of life [20], demonstrating that increased glucose uptake alone is sufficient to break immune tolerance. Thus, data from experiments that directly modulate enzyme activity, without broad influences on signaling network, are more informative for dissecting the causal relationship between metabolism and T cells. Nevertheless, the activity of some key signaling proteins, such as kinases, acetylases, and methylases, is controlled by metabolic co-factors, such as ATP, acetyl coenzyme A (acetyl-CoA), and S-adenosyl-l-methionine (SAM) [21]. Thus, metabolism can actively control cellular signaling. In these cases, metabolites are in the driver’s seat instead of acting merely as downstream events of signaling.

### Specificity

T-cell responses are highly diverse to different immune challenges. For instance, naïve CD4^+^ T cells can differentiate into distinct effector subsets, including T helper 1 (Th1), Th2, Th17, and peripheral regulatory T (pTreg) cells [22]. Unlike lineage-specifying transcription factors that are selectively expressed in certain Th subsets, the expression of most metabolic enzymes is unlikely limited to certain T-cell subsets. Initial observations from *in vitro* studies showed that effector T cells (Th1, Th2, and Th17) favor glycolysis, while Treg cells prefer OXPHOS [23]. Later studies showed that effector T cells also engage in OXPHOS [24] and that glycolysis is also important for Treg cell induction [25]. Thus, how the evolutionarily conserved metabolic pathways regulate diverse T-cell responses at high specificity is still a puzzle. For example, acetyl-CoA, a central metabolite in carbon metabolism, promotes T-cell differentiation though acetylation of key loci associated with T effector functions, such as those encoding the inflammatory cytokines interferon-γ (IFN-γ) and interleukin-17A (IL-17A) [26–28]. How the universal metabolite acetyl-CoA acetylates selected loci in certain T-cell subsets remains to be determined. The specificity question is not unique in immunometabolism, but a general one in the metabolism-epigenetics field [21].

### Chemical inhibitor vs. genetic manipulation

Small molecule inhibitors that block metabolic enzyme activities are widely used in metabolism research. However, small molecule compounds often have off-target effects, and data from chemical inhibitor experiments need to be backed up with other experimental approaches, such as genetics. For instance, etomoxir has often been used as an inhibitor of carnitine palmitoyltransferase 1A (CPT1A) to study the function of long-chain fatty acid oxidation in immunometabolism. However, T-cell phenotypes from studies using etomoxir were not observed in mice with T cell-specific deletion of CPT1A [29]. In fact, etomoxir can function as a prodrug that consumes CoA during metabolic conversion to etomoxiryl-CoA, and it is the consumption of cellular CoA (not the inhibition of CPT1A *per se*) that accounts for the phenotypes observed in etomoxir-treated macrophages [30]. Whether this is the case in T cells warrants further investigation. Thus, although chemical inhibitors facilitate immunometabolism research and hold potential for immunotherapy, we should be cautious about the interpretation of data from inhibitor studies alone.

Bearing these important considerations of immunometabolism in mind, we will discuss the roles of major metabolic pathways along the life journey of T cells, including T-cell development and homeostasis in the immunological steady state, as well as during T-cell activation, expansion, differentiation, contraction, and memory formation in response to immune challenge. We will also discuss targeting T-cell metabolism for disease therapy and potential future directions of this field. Unless otherwise specified, T cells mentioned in this review refer to T-cell receptor (TCR) αβ^+^ T cells, including CD4^+^ Th cells and CD8^+^ cytotoxic T cells.

## The life journey of T cells

The classic life journey of T cells includes development in the thymus, homeostasis of naïve T cells in the periphery, and antigen-driven clonal expansion and differentiation, followed by contraction and memory formation. In the thymus, T cells develop from common lymphoid progenitors generated by hematopoietic stem cells (HSCs). After egress from the thymus, mature naïve T cells are maintained in secondary lymphoid organs in a quiescent state. Upon recognition of a cognate antigen in the presence of appropriate co-simulation signals, naïve T cells become activated and undergo extensive clonal expansion accompanied by differentiation into effector cells. During acute infections, T-cell expansion peaks about one week after infection, which is followed by massive clonal contraction during which ~95% of effector T cells die. T cells that survived the contraction phase become memory T cells, which mediate an enhanced T-cell response upon reinfections.

## Metabolic control of T-cell development

The life journey of T cells begins with their development in the thymus. Development of the αβ T-cell lineage is a tightly regulated process, and the transcriptional regulation of T-cell development has been well characterized [31]. From a cell biology perspective, double-negative (DN) thymocytes undergo rapid proliferation after β-selection [32], which may have a high demand for nutrients and cell anabolism. However, the metabolic control of early T-cell development remains largely unexplored, as CD4-Cre transgenic mice, the most commonly used model to conditionally target genes of interest in T cells, delete target genes at the double-positive (DP) stage. For instance, depletion of GLUT1 with CD4-Cre does not affect T-cell positive selection, while depletion of GLUT1 at DN thymocytes with LCK-Cre causes reduced numbers of DP and CD4 single-positive (SP) T cells [33], revealing that GLUT1-mediated glucose uptake contributes to T-cell development. Glucose has multiple metabolic fates in cells, and it is unclear how glucose metabolism affects T-cell development. The roles of other metabolic pathways in T-cell development remain to be determined with appropriate mouse models.

## Metabolic control of naïve T-cell homeostasis

After development in the thymus, mature T cells enter the circulation and home to secondary lymphoid organs as naïve T cells. The state of naïve T cells is maintained by TCR signaling from self-peptide major histocompatibility complexes and the cytokine IL-7 [34]. Unlike quiescent states of other mammalian cells, such as HSCs, naïve T cells continuously survey the blood and lymphoid system for the presence of exogenous antigens. Thus, metabolic requirements of naïve T cells are likely different from that of other non-circulating quiescent cells. In support of this notion, aerobic/anerobic glycolysis mediated by the metabolic enzyme lactate dehydrogenase A (LDHA) is important for the homeostasis of HSCs but not naïve T cells [27, 35]. Similarly, although GLUT1 contributes to T-cell development in the thymus, it is not required for the survival of naïve T cells in the periphery [33]. Deletion of CPT1A also does not affect naïve T-cell homeostasis [29], indicating that long-chain fatty acid oxidation is also dispensable for such homeostatic control. These findings suggest that naïve T cells may utilize another metabolic pathway, or engage redundant metabolic pathways, for the maintenance of homeostasis.

## Metabolic control of T-cell activation

There is a well-defined lag between antigen-induced activation of naïve T cells and the first division of activated T cells (~24 h−36 h), during which T cells transit from the quiescent state to a rapid proliferating state. During this activation phase, thousands of genes are up- and down-regulated to prepare T cells for rapid cell division and differentiation, which have been extensively studied at transcriptional and post-transcriptional levels [36]. This special phase of T cells activation may have different metabolic requirements from the ensuing cell proliferation phase. For example, mitochondrial reactive oxygen species (mROS) produced after TCR ligation appears to be critical for T-cell activation in terms of activation of the nuclear factor of activated T cells and production of the T-cell growth factor IL-2, but it is not required for lymphopenia-driven T-cell proliferation [37]. These conclusions are based on the observation that T-cell activation is inhibited in the absence of the mitochondrial complex III subunit Rieske iron sulfer protein that promotes mROS production [37]. However, it is unknown whether additional electron transport chain-dependent activities, such as OXPHOS, and promotion of redox balance support T-cell activation. Mitochondrial metabolism can also promote T-cell activation through the induction of a one-carbon metabolism pathway, which is additionally required for nucleotide synthesis and DNA replication [38]. Upregulation of the T-cell activation markers CD69 and CD25 appears normal in the absence of glucose [37]. However, recent studies have shown that glycolytic ATP is required for optimal T-cell activation, as marked by CD62L downregulation, by promoting phosphatidylinositol-3-kinae (PI3K)-serine/threonine kinase (AKT) signaling [28, 39], which phosphorates and inactivates the forkhead box protein O (Foxo) family of transcription factors that enforce T-cell quiescence [40–42].

## Metabolic control of T-cell expansion

Due to a large repertoire of TCRs, the absolute number of naïve T cells specific for any given antigen is low [43]. There are two types of T-cell expansion with different metabolic requirements. The classic clonal expansion of T cells followed by pathogen infection is usually accompanied by acute inflammation, which generates the required number of effector T cells to effectively combat the rapidly replicating pathogens. This life or death situation may explain why activated T cells are among the fastest proliferating cell types in mammals. Another kind of T-cell expansion is homeostatic proliferation, which is relatively slow and usually driven by lymphopenia, either physiologically during the neonatal period or pathogenically [44, 45]. The majority of immunometabolism studies are focused on the classic clonal expansion of T cells. However, metabolic pathways essential for T cell clonal expansion can be dispensable for homeostatic proliferation of T cells. For instance, LDHA-mediated glycolysis is essential for optimal clonal expansion of T cells in models of bacterial and viral infections or in a model of antigen-driven experimental autoimmune encephalomyelitis [28, 39], while it is not required for homeostatic proliferation of T cells or expansion of autoreactive T cells [27]. Thus, the type of inducer and the rate of cell division may determine the metabolic requirements of expanding T cells.

*In vitro* assays with chemical inhibitors showed that either glycolysis or OXPHOS is sufficient to fuel T-cell expansion [16]. However, recent *in vivo* studies revealed that glycolytic production of ATP promotes robust T cell clonal expansion by fueling the PI3K-AKT-Foxo pathway [28, 39]. Recent genome-wide CRISPR screening of T-cell fitness genes also revealed that, although mitochondrial Complex I and Complex V components are dispensable for T-cell expansion *in vitro*, as reported [16, 46], they are essential for T-cell expansion *in vivo* [46]. Microenvironment differences may explain the conditional essentiality of glycolysis and OXPHOS in T-cell expansion *in vitro*. One major environmental factor is oxygen, the ultimate receptor for electrons removed from nutrients during energy-generating catabolism. The oxygen concentration in organs and tissues is far below those used in normal laboratory incubators (~21%), and inflammation is known to cause hypoxia in lymphoid organs and tissues [47]. Thus, oxygen is a limiting factor for OXPHOS *in vivo*, which may explain why OXPHOS could support T-cell expansion without aerobic glycolysis *in vitro* but not *in vivo* [46]. Another important environmental factor is the concentration of nutrients in the culture medium [48]. The commonly used RPMI 1640 medium for T-cell culture has abundant glucose and amino acids, but both can be limiting *in vivo*, especially in inflamed tissues. Thus, supraphysiological levels of oxygen and nutrients used in T cell cultures may account for the ‘loose’ metabolic requirements of T-cell expansion *in vitro*.

In addition to energy-generating cell catabolism, proliferating T cells require building blocks for biosynthesis, which are derived from anabolic pathways. Cells need to replicate the entire genome before cell division; thus, nucleotide synthesis is critical for rapid T-cell proliferation. Biosynthesis of nucleotides requires carbon from glucose and nitrogen from amino acids. Glucose-dependent serine and nucleotide synthesis are important for CD8 T-cell expansion *in vivo*, especially under serine-restricted conditions [49, 50]. In line with these studies, the one-carbon metabolic enzyme methylenetetrahydro-folate dehydrogenase 2 (MTHFD2), which regulates *de novo* purine synthesis, also contributes to T-cell expansion [51].

Lipids are net-synthesized in proliferating cells. Thus, a dispensable role for long-chain fatty acid oxidation in T-cell expansion is expected [29]. Whether middle- and/or short-chain fatty acid oxidation regulates T-cell expansion remains unknown. In contrast, key enzymes involved in lipid synthesis, such as acetyl-CoA carboxylase 1 and their transcriptional regulators sterol regulatory-element binding proteins, are critical for T-cell expansion [52–54].

In summary, it seems that the metabolic requirements of T cell clonal expansion are similar to that of other proliferating mammalian cells [55, 56]. T cells likely employ the highly efficient metabolic network that have evolved to support such rapid cell proliferation. As far as we know, there is no metabolic pathways or enzymes that are uniquely required for proliferating T cells but not involved in the proliferation of other cell types. As such, proliferating T cells represent an ideal model to study metabolic requirement of rapidly proliferating mammalian cells.

## Metabolic control of T-cell differentiation

Activated CD4^+^ Th cells can differentiate into distinct effector subsets [22]. An early report showed that the T cell co-stimulatory receptor CD28 drives glycolysis upon T-cell activation [57]. However, CD28 signaling-induced glycolysis far surpasses the needs of ATP production and macromolecule biosynthesis [57]. In certain infection and autoimmune settings, CD28 signaling is not essential for T-cell expansion, but is required for cytokine production [58, 59], suggesting excessive glucose uptake in proliferating T cell may support differentiation in addition to cell proliferation. Of note, effector Th cells favor aerobic glycolysis while Treg cells prefer fatty acid oxidation [23], which has spurred interest in the study of the metabolic regulation of Th cell differentiation.

**Figure 2:**
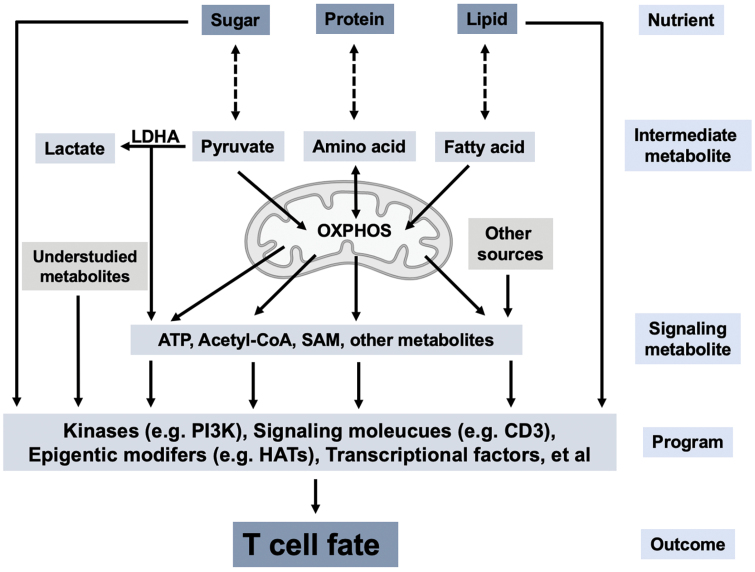
Mechanisms of metabolic control of T-cell fate. The interconversions between major nutrients (sugar, protein, and lipid) and intermediate metabolites (pyruvate, amino acid, and fatty acid) during catabolism and anabolism generate energy and building blocks, respectively, for T cells. Certain metabolites, such as ATP, acetyl-CoA, and SAM, function as signaling molecules to modulate the activity of key proteins involved in T-cell fate decision, such as kinases and HATs. This metabolite-signaling model partially explains how metabolism controls T-cell fate. LDHA, lactate dehydrogenase A; OXPHOS, oxidative phosphorylation; ATP, adenosine triphosphate; SAM, S-adenosyl-l-methionine; HAT, histone acetyltransferase.

An emerging mechanism of metabolic regulation of Th cell differentiation is metabolic-epigenetic regulation (Fig. 2). Selective expression of lineage-defining genes in activated Th cells shapes T-cell differentiation [22]. The expression of these key genes requires epigenetic modification of histones and/or DNA at relevant genomic loci. Many enzymes involved in epigenetic modifications use metabolites as co-factors, thus connecting metabolism and gene expression [60]. Several such metabolites have been reported to regulate Th cell differentiation, including acetyl-CoA and SAM. An early study showed that ATP-citrate lyase (ACLY), the enzyme converting citrate into acetyl-CoA, is required for histone acetylation during fibroblast differentiation *in vitro* [61]. Using LDHA-deficient T cells as a model, aerobic glycolysis has been shown to promote Th1 and Th17 cell differentiation in part through such a metabolic-epigenetic pathway [27, 28]. In this setting, the defect of Th1 cell differentiation of LDHA-deficient cells can be rescued by acetate, another acetyl-CoA donor, demonstrating a key role for acetyl-CoA in Th cell differentiation [27]. In line with these findings, a recent study of GLUT3 in T cells also showed that the GLUT3-glycolysis-acetyl-CoA-acetylation pathway is important for Th17 cell differentiation [26]. Similarly, methionine was reported to regulate Th17 cell differentiation through histone methylation by SAM, the sole donor of methyl groups for methylation reactions [62]. These studies demonstrate that metabolism can modulate T-cell differentiation by providing key metabolites involved in epigenetics regulation.

In addition to the metabolite-epigenetics axis, metabolism can also affect T-cell differentiation through metabolite-signaling pathways. Except for the glycolytic ATP-PI3K axis mentioned above, the glycolytic metabolite phosphoenolpyruvate has been shown to promote Th1 and CD8 T-cell differentiation by enhancing calcium signaling via sarco/endoplasmic reticulum Ca^2+^-ATPase (SERCA) [63]. In addition, calcium enhances TCR signaling by inducing the dissociation of CD3 from the charged membrane lipid [64]. Similarly, increasing plasma membrane cholesterol content through inhibition ACAT1, a key cholesterol esterification enzyme, potentiates effector function, and enhances proliferation of CD8^+^ T cells via boosting TCR signaling [65, 66].

Based on their routes of differentiation, Treg cells can be classified as tTreg cells that develop in the thymus and pTreg cells that are differentiated from naïve T cells in peripheral tissues [67]. As pTreg cells can be induced from naïve CD4 T cells *in vitro*, most metabolic studies of Treg cells have focused on pTreg cells. Both glycolysis and OXPHOS are active in these cells [23, 68]. Although glycolysis promotes pTreg cell proliferation, it blocks the immune repressive activity of these cells via unknown mechanisms [68]. Foxo1 has been shown to promote Treg cell-mediated immune tolerance [69], it is thus possible that glycolysis may repress Treg cell function through the recently reported glycolytic ATP-PI3K-AKT-Foxo1 signaling axis [28, 39].

Although mitochondrial metabolism is reported to be important for the suppressive activity of pTreg cells [23, 70, 71], the underlying mechanisms remain unclear. Similarly, both lipid uptake and lipid synthesis are selectively important for the fitness of pTreg cells in the tumor environment but not in other tissues [72, 73], suggesting context-dependent roles of lipid synthesis in pTreg cells. Importantly, the cause and consequence of pTreg cell differentiation and metabolism reprogramming is still to be determined. It has been shown that forced expression of forkhead/winged helix transcriptional factor P3 (FoxP3), the linage-specifying transcription factor of Treg cells, represses glycolysis and induces oxidative metabolism in T cells [68]. Considering overexpressing of Foxp3 alone is sufficient to confer conventional T cells with immune suppressive activity [74, 75], it is important to investigate whether the metabolic features of pTreg are the cause or consequence of Foxp3 induction and pTreg cell differentiation. Unlike pTreg cells, metabolic regulation of tTreg development is poorly studied. The initial observation of pTreg dependence on long-chain fatty acid oxidation was not observed in natural Treg (nTreg) cellsin CPT1A-deficent mice [29], and LDHA-mediated glycolysis also does not affect development of tTreg cells [27].

Although various mechanisms have been uncovered, how metabolic regulation of T-cell differentiation achieves specificity remains to be determined. For example, in various models of metabolite-epigenetic pathways, how the universal metabolites selectively modulate certain gene loci is not fully understood. One possibility is that these epigenetic modifications need to co-operate with distinct transcription factors that recruit histone modifying enzymes to different gene loci.

## Metabolic control of T-cell contraction

Following clonal expansion, most effector T cells die during the contraction phase and the surviving T cells become memory T cells. It appears that, except for the combined deficiency of Bim and Fas [76–78], two key proteins promoting apoptosis, no other proteins have been shown to delay or prevent T-cell contraction. However, cell death is likely the consequence but not the cause of T-cell contraction. The life and death choice of T cells during the contraction phase is an important but understudied field. As metabolism is the fundamental feature of all living cells, deciphering the metabolic basis of T-cell contraction is an interesting direction.

## Metabolic control of T-cell memory

A defining feature of adaptive immunity is immunological memory. The cellular basis of immunological memory is the formation of long-lived memory T cells and B cells. In spite of extensive investigations [79], the mechanisms of memory T-cell differentiation and maintenance are still poorly understood. Although many factors, including transcription factors T-cell factor 1 (TCF1) and Foxo1, are necessary [80–83], none is sufficient to induce memory T-cell formation. For metabolic processes, lipid metabolism has been substantially explored in memory T cells. Earlier experiments with chemical inhibitors and short hairpin RNA (shRNA)-mediated genetic knockdown showed that lipid uptake and metabolism, particularly CPT1A-mediated long-chain fatty acid oxidation, were important for the differentiation and maintenance of both central memory T cells [84, 85] and resident memory T cells [86]. Similarly, IL-7 has been shown to promote memory T-cell survival in part through induction of glycerol transport and fatty acid oxidation [87]. However, later studies with T cell-specific deletion of CPT1A, the key gene responsible for importing long-chain fatty acids into mitochondria, do not support a critical role of CPT1A in memory T cells [29]. In fact, whether and how mitochondria metabolism promotes memory T-cell differentiation and/or survival remain largely unknown. Production of ATP by OXPHOS was proposed to be a mechanism to explain memory T-cell survival [87], but why other nutrients, including glucose and amino acids, cannot replace fatty acids for ATP production is unclear. For example, pyruvate metabolism is also reported to be important for memory T cells [88]. The role of glycolysis in control of memory T-cell responses appears to be dependent on memory T-cell subsets. While inhibition of glycolysis during T-cell activation promotes central memory T-cell differentiation [89], enhanced glycolysis in T cells deficient in the von Hippel-Lindau tumor suppressor (VHL) results in enhanced effector memory T cell differentiation [90]. The underlying mechanisms of glycolytic regulation of memory T-cell responses remain obscure. Besides glycolysis and fatty acid oxidation, the roles of other metabolic pathways in memory T cells need to be explored.

## Targeting T-cell metabolism to treat immunological disorders

Targeting metabolism has been explored for the treatment of immune-related diseases. Methotrexate (MTX), an antimetabolite that blocks DNA synthesis via inhibition of dihydrofolate reductase, is used clinically for the treatment of autoimmune or inflammatory diseases, such as rheumatoid arthritis. We discussed little about DNA metabolism in our review, because it is essential in all cells. Due to severe side-effects, MTX is only prescribed for patients who do not respond to other anti-inflammation treatments.

A priority in developing therapeutics targeting immunometabolism is therefore the need for specificity. To this end, we may learn from the cancer metabolism field. Although extensive investigations over the past decades have generated a rich knowledge about metabolic regulation of cancer cells [91], which is considered a cancer hallmark [92], targeting cancer metabolism beyond nucleotide synthesis is still highly challenging [93]. One exception is the small molecule inhibitors ivosidenib and enasidenib, which target mutated isocitrate dehydrogenase (IDH) and have been approved for the treatment of solid and hematologic malignancies with such mutations [94]. However, this success of targeting cancer metabolism is more of an exception than a rule. Mutated IDH1 and IDH2 gain neomorphic enzyme activity that is not present in wild-type IDH; thus, inhibitors can be developed to specifically target mutant enzymes. In most cases, metabolic differences between cancer cells and normal cells are mostly quantitative, not qualitative. Searching for an appropriate window to inhibit cancer cell metabolism without significantly impacting normal cells is still a challenge.

The same problem exists when we wish to target T-cell metabolism (or immunometabolism in general) to treat diseases. However, targeting immunometabolism might be relatively “easier” than targeting cancer cell metabolism due to different objectives. For cancer treatment, the goal is to kill cancer cells or at least to completely inhibit cancer cell growth, otherwise the cancer will progress. For immune-related diseases, we do not have to kill pathological immune cells to treat diseases. For example, LDHA-deficient autoreactive T cells can still proliferate to the same extent as wild-type autoreactive T cells in scurfy mice [27], suggesting that LDHA is unlikely a good target to treat T-cell lymphoma or other cancers. However, LDHA-deficient autoreactive T cells have much lower expression of IFN-γ and other inflammatory cytokines, which extends the lifespan of scurfy mice from about 4 weeks to more than 1 year [27], suggesting that LDHA is a potential target for the treatment of autoimmune and inflammatory diseases. Downstream of LDHA and glycolysis, inhibition of ACLY also represses effector T-cell differentiation [26, 27], suggesting that acetyl-CoA metabolism represents a potential target as well. Particularly, an ACLY inhibitor has been approved for the treatment of hypercholesterolemia [95]. Thus, compounds originally developed to target cancer metabolism or other metabolic diseases may be repurposed to target immunometabolism. Nevertheless, we still need to explore whether the metabolic difference between immune cells and other normal cells is big enough to provide a therapeutic window.

In contrast to inhibiting T-cell metabolism to treat autoimmune, inflammatory, and allergic diseases, boosting T-cell metabolism may be used to improve T-cell function in anti-tumor and anti-infection settings. To achieve this goal, we need to know which metabolic pathway is limiting to drive T-cell responses *in vivo*. It has been proposed that metabolic competition between cancer cells and immune cells drives T-cell dysfunction via glucose deprivation in the tumor microenvironment [96]. A recent study showed that metabolic features of cancer cells and tumor infiltrating T cells (TILs) are programmed via cell-intrinsic mechanisms, with limited influence from the tumor microenvironment [97]. In fact, T cells take up more glucose than cancer cells in the tumor microenvironment, while cancer cells prefer glutamine as a nutrient source [97]. In support of this notion, inhibition of glutamine metabolism blocks cancer cell metabolism and at the same time boosts T-cell function, which promotes anti-tumor immunity [98]. This is an excellent example of exploring metabolic differences between T cells and cancer cells to metabolically enhance T-cell function. Nevertheless, whether other normal cells can tolerate the inhibition of glutamine metabolism remains to be determined. Thus, a major hurdle of targeting immunometabolism remains the degree of specificity, whether it is inhibiting or enhancing T-cell metabolism.

One strategy to circumvent the specificity issue is to combine metabolic manipulations with adoptive T-cell therapy. Overexpression of metabolic enzymes in T cells, such as phosphoenolpyruvate carboxykinase 1 (PCK1), has been shown to bolster T-cell functions and enhance the efficacy of adoptive T-cell therapy in mouse tumor models [63]. Further studies are required to explore potent but safe metabolic pathways to manipulate T cells.

## Future perspective

As an emerging sub-field of immunological research, immunometabolism has drawn substantial attention in recent years. In the T-cell field, the classic three-signal model (antigen, co-stimulation, and cytokine) provides a framework to explain most T-cell activities. For metabolic regulation of T cells (and immunometabolism in general), a unifying framework to explain immunometabolic behaviors of T cells is still lacking. For example, mitochondrial metabolism is generally believed to be important for T-cell stemness and longevity, which is counterintuitive since long-lived HSCs engage glycolysis to maintain stemness, likely to reduce ROS production [35]. From an evolutionary point of view, T cells may co-opt the highly conserved metabolic network to support their functions. It is unlikely that certain metabolic pathways have evolved to specifically support the needs of T cells (or other immune cells), which appeared long after the evolution of current metabolic networks.

In the field of immunometabolism, several important questions may be addressed in the coming years. First of all, among >3000 metabolic enzymes [99], how many are involved in clonal expansion of T cells? As mentioned in the Introduction, metabolic studies are historically dominated by cancer cells (proliferating) or organ extracts (post-mitotic and non-proliferating). Metabolic features of physiological proliferating cells are usually deduced from cancer cells, but lack direct evidence. T cells are one of the few cell types in adult mammals that can rapidly proliferate both *in vitro* and *in vivo*, which offers an opportunity to study the metabolic basis of normal cell proliferation. So far, most research has focused on well-studied pathways, accounting for a small portion of metabolic enzymes. A systematic investigation of the functions of metabolic enzymes in proliferating T cells will validate old insights into the metabolic requirements of cell proliferation, while generating new ones. Second, as metabolism supports and regulates all cellular processes, are there distinct features of T-cell metabolism? Do activated T cells require unique metabolic activities that are largely dispensable in other cell types? For instance, the nonessential amino acid threonine and the related threonine dehydrogenase are uniquely required for the proliferation of embryonic stem cells, but are dispensable for other cell types [100]. Further identification of T cell-specific metabolic pathways may lead to novel therapeutic targets with high specificity. Finally, unlike lineage-specifying factors that are necessary and sufficient to change cell fate, such as Foxp3 for Treg cells and Yamanaka factors for induced pluripotent stem cells, whether we could change T-cell fate through modulation of metabolism remains to be demonstrated.
